# The effect of staphylococcal infection on cathelicidin and β-defensin mRNA levels in epithelial cells lining the mammary gland cistern

**DOI:** 10.3389/fimmu.2026.1804785

**Published:** 2026-05-14

**Authors:** Emilia Bagnicka, Adrianna Szprynca, Ewa Kościuczuk, Justyna Jarczak, Magdalena Rzewuska, Tomasz Sakowski, Magdalena Zalewska

**Affiliations:** 1 Institute of Genetics and Animal Biotechnology, PAS, Jastrzębiec, Poland; 2 Laboratory of Regenerative Medicine, Medical University of Warsaw, Warsaw, Poland; 3 Department of Preclinical Sciences, Institute of Veterinary Medicine, Warsaw University of Life Sciences, Warsaw, Poland; 4 University of Warsaw, Faculty of Biology, Institute of Microbiology, Department of Bacterial Physiology, Warsaw, Poland

**Keywords:** antimicrobial peptides, dairy cattle, mRNA, subclinical mastitis, udder

## Abstract

**Introduction:**

The bovine mammary gland employs epithelial defenses against bacterial invasion, including a bilayer columnar epithelium lining the lactiferous sinuses and mucosal folds at the inner end of the teat canal. The aim of this *in vivo* study was to compare mRNA transcript levels of selected β-defensins and cathelicidins in mammary gland cistern lining epithelial cells (MGCLEC) derived from the whole healthy udders and those naturally infected with coagulase-positive (CoPS, *Staphylococcus aureus*) or coagulase-negative staphylococci (CoNS, several strains).

**Methods:**

The expression of selected genes was analyzed using RT-qPCR. Followed by: 'TATA box-binding protein (TBP) and hypoxanthine phosphoribosyltransferase1 (HPRT1) were used as reference genes.

**Results and discussion:**

No expression of *TAP*, *CATHL4* or *CATHL6* was detected in the tested samples. For all other genes, a significant interaction between infection status and parity was observed. While *BNBD1* expression remained low, *BNBD4* showed the highest transcription levels among defensins, particularly in older cows (lactations 3–4). Significant upregulation of *BNBD5* was noted in the CoPS (*S. aureus*) group compared to CoNS. *LAP* transcription was significantly higher in CoPS (*S. aureus*) infected quarters than in CoNS and healthy groups across both parity classes. Additionally, *CATHL5* expression was elevated in younger CoPS (*S. aureus*) infected cows compared to the CoNS and older CoPS (*S. aureus*) groups. Our results suggest that *BNBD4*, *BNBD5*, and *CATHL5* are key transcriptional components of the MGCLEC innate immune response. Conversely, the absence of *TAP*, *CATHL4*, and *CATHL6* transcripts indicates that these specific peptides are not upregulated in cistern epithelial cells during the subclinical stage of staphylococcal infection.

## Introduction

Milk from cows with clinical or sub-clinical mastitis may contain high levels of bacterial pathogens. Despite pasteurization, these bacteria can threaten human health (1): some produce thermostable toxins that remain active even though bacteria are eliminated. For instance, coagulase-positive staphylococci (CoPS), such as *Staphylococcus aureus*, produce enterotoxins causing food poisoning. Although coagulase-negative staphylococci (CoNS) are typically opportunistic and less virulent than CoPS, they are implicated in various conditions including toxic shock syndrome (2).

Structural defenses of mammary gland include the lactiferous sinuses lined with a bilayer columnar epithelium, and milk ducts lined with a stratified cuboidal or columnar epithelium. The primary barrier is the teat canal, which is maintained by the layered structure of sphincter muscles. Its mucosal folds cover the canal during udder filling thereby preventing the entry of pathogen (3). Furthermore, keratin within the canal acts as both physical and chemical barrier against invasion (3, 4).

A second defense line includes polymorphonuclear leukocytes, macrophages and epithelial cells which produce antimicrobial proteins or peptides (AMPs), including cathelicidins and defensins (5). These broad spectrum peptides play a critical roles in various tissues (6, 7). Importantly, both families function in an oxygen-independent manner remaining active under the hypoxic conditions of infected tissues (8). They activity against mastitis pathogens is well-documented (9–13).

AMP expression in bovine mammary gland tissues has been studied (9, 10, 14–16), mostly using *in vitro* cell cultures (4, 17, 18). *In vivo* reports on natural infections, remain scarce (9, 14, 19). Furthermore, research primarily focuses on the secretory epithelial tissue due to its role in milk production (9, 14, 15). Chronic intramammary infections cause more significant milk composition changes than acute cases (20). Notably, milk decreases can precede clinical signs by up to one week (21), unless the infection is eliminated.

Consequently, transcript levels of AMP genes in udder cisternal lining epithelium cells (MGCLECs) from pathogen-free *vs*. infected tissues remain limited. The role of MGCLECs in mammary pathophysiology is also not fully understood. Beyond acting as a mechanical barrier, this tissue participate in immune responses. Zalewska et al. (22) reported similar expression of acute phase protein genes (serum amyloid A3 (*SAA3*), haptoglobin (*HP*) and ceruloplasmin (*CP*)), in both MGCLECs and mammary secretory epithelial cells (MECs). Additionally, certain bacteria persist by adhering to and damaging the teats and milk cistern lining epithelium (23).

This *in vivo* study aimed to determine β-defensin and cathelicidin transcript levels in MGCLECs from bovine mammary glands naturally infected with CoPS (*S. aureus*) or CoNS, compared with healthy udders.

## Material and methods

### Animals, sampling, and microbiological analysis

Milk and tissue samples were collected from 40 Polish Holstein-Friesian dairy cows, Black and White variety, born and reared in a herd located in Central Poland. Animals were loose-housed with *ad libitum* water access and fed a total mixed ration (TMR) based on corn silage (75%), concentrates (20%) and hay (5%) supplemented with VITAMIX KW mineral and vitamin mixture (Polmass, Bydgoszcz, Poland). Feeding followed standards developed by the *Institut National de la Recherche Agronomique* (INRA, France) and adopted by the National Research Institute of Animal Production (IZ PIB, Poland), with approx. 5% feed refusal (24).

Milk herd yield was 8, 670 kg milk per lactation (4.02% fat, 3.45% protein). Milking was performed twice a day in a herringbone milking parlor (DeLaval, Tumba, Sweden). Cows being between their first to fourth lactation and were culled due to reproduction issues or chronic, recurrent mastitis. At slaughter, the animals were asymptomatic, showing no clinical signs of mastitis or lameness. Experimental groups included cows with chronic, recurrent infection caused by *S. aureus* (CoPS) or CoNS which had proven refractory to antibiotic treatments. To ensure a homogenous study population of chronic inflammatory states, clinical history was not included as a separate variable. To comply with commercial meat standards, all animals underwent at least a one-month antibiotic withdrawal period prior to sampling.

Animals were slaughtered in a registered slaughterhouse at the late lactation (286 ± 25 days) *via* mechanical stunning and exsanguination, following national standards. Mammary gland samples were collected from the prepared udder immediately post-slaughter in a dedicated room with permission from the slaughterhouse owner and supervising veterinarian.

Briefly, MGCLEC samples were collected from the area surrounding the teat opening to the gland cistern from each quarter. Samples were rinsed in ice-cold phosphate buffered saline (PBS, pH~7.2; Merck), snap-frozen in liquid nitrogen, and stored at -80 °C.

Two days before slaughter, two milk types were collected per quarter to assess udder health. Foremilk (20 mL) for microbiological screening was sampled aseptically before evening milking after discarding the first three streams into the pre-milking unit and disinfecting teats with 70% ethanol. These samples were stored at 4°C until further analysis. For composition analysis, milk was collected using a mechanical quarter milker, preserved with Microtabs (Bentley Instruments, Chaska, MN, USA) and also stored at 4 °C. SCC was determined *via* IBCm analyzer (Bentley Instruments, Chaska, MN, USA), while lactose content *via* Fossomatic FT2 (FOSS, Hillerød, Denmark).

The following day, milk samples for microbiology were allowed to reach room temperature. Then, 100 μL of each thoroughly mixed sample was streaked onto Columbia agar with 5% sheep blood, Chapman–Mannitol Salt Agar (MSA), and MacConkey agar (bioMérieux, Craponne, France). Plates were incubated at 37 °C for 24 to 48 hours. Representative colonies were then subcultured for pure bacterial strain isolation.

Isolates were identified *via* colony/cell morphology and biochemical properties assessment. Gram-negative rods were identified using the API 20E test (bioMérieux, Craponne, France). Gram-positive were differentiated using the catalase test into *Staphylococcus* spp. and *Micrococcus* spp. (catalase-positive) and *Streptococcus* spp. and *Enterococcus* spp. (catalase-negative).

Staphylococcal coagulase production was assessed *via* tube test (rabbit plasma 1:5; Biomed, Warsaw, Poland) at 1, 3, 6, and 24 hours (25, 26). Isolates failing to coagulate plasma after 24 h were classified as CoNS, positive results were categorized as *S. aureus*, namely CoPS, which was further confirmed *via* API Staph and Slidex Staph kits (bioMérieux). The most frequent CoNS species was *S. epidermidis*. Other identified species included *S. sciuri*, *S. vitulinus*, *S. xylosus*, *S. chromogenes*, and *S. lentus*. All CoPS isolates were *S. aureus*, hereafter referred to as CoPS (*S. aureus*).

Quarters with mixed infections (e.g., CoPS (*S. aureus*) and CoNS, or staphylococci and streptococci) were excluded. Samples containing Gram-negative bacteria (N = 3) were also omitted from the study.

Mammary gland health was determined *via* microbiological status, SCC, and lactose content. Healthy quarters were defined by a negative cultures and SCC < 2 x 10^5^ cells/mL (International Dairy Federation (IDF) standards) (27). The lactose threshold was set at 4.7% with higher values indicating healthy quarters (28). Clinical and lactation histories were verified using the electronic herd management database.

Preliminary analyses (MANOVA and GLM; SAS/STAT 14.3, 2002–2012, ver. 9.4) showed no significant differences in gene expression between lactations 1 and 2 or between lactations 3 and 4 (p > 0.05). Consequently, parity was merged into two parity classes, *viz.* lactation 1, 2 and lactation 3, 4.

From 160 initial MGCLEC samples, 62 were selected for analysis. The healthy control group (H) comprised quarters from whole bacteriologically negative udders with low SCC and lactose level>4.7%. Quarters with acute mastitis, mixed infections, or non-staphylococcal pathogens (e.g., *Escherichia coli, Streptococcus* spp.) were excluded. To ensure independence observations, a maximum two quarters per cow was included. Six groups were distinguished: two control groups, i.e. in lactation 1-2 (H-1, 2; N = 9) and lactation 3-4 (H-3, 4, N = 9); two groups infected with CoPS (*S. aureus*), i.e. in lactation 1-2 (CoPS-1, 2, N = 14) and lactation 3-4 (CoPS-3, 4, N = 14), and two groups infected with CoNS, i.e. in lactation 1-2 (CoNS-1, 2, N = 7) and lactation 3-4 (CoNS-3, 4, N = 9).

### RNA isolation and reverse transcription quantitative polymerase chain reaction analyses

Total RNA was isolated using the RNeasy Mini Kit (Qiagen, Germany). RNA quantity and integrity were determined *via* NanoDrop 2000 (Thermo Fisher Scientific, Waltham, Massachusetts, USA), and Bioanalyzer 2100 using the RNA 6000 Nano LabChipKit (Agilent Technologies, Santa Clara, USA), respectively. Only samples with >50 ng RNA, A260/280 and A260/230 ratios of ~2.0, and a RNA Integrity Number (RIN) > 7.5 were analyzed.

Total RNA (0.5 µg) was reverse-transcribed using the Transcriptor First Strand cDNA Synthesis Kit (Roche, Basel, Switzerland). 50 µM oligo(dT) primers. The 20 µl reaction mixture included 13 µl of RNA, 4 µl of reverse transcriptase buffer, 2 µl of 10 mM deoxynucleotides (dNTPs), 0.5 µl of protector RNase Inhibitor (40 U/µl), and 0.5 µl of reverse transcriptase (20 U/µl). Incubation was performed at 50 ˚C for 60 min, followed by enzyme inactivation at 85 ˚C for 5 min. The resulting cDNA was stored at -20 ˚C.

Six potential housekeeping genes (HKGs) were evaluated: Actin Beta (*ACTB*), Glyceraldehyde-3-Phosphate Dehydrogenase (*GAPDH*), HOX Transcript Antisense RNA (*HPRT1*), Succinate Dehydrogenase Complex Flavoprotein Subunit A (*SDHA*), TATA-Box Binding Protein (*TBP*), and Tyrosine 3-Monooxygenase/Tryptophan 5-Monooxygenase Activation Protein Zeta (*YWHAZ*). Primers for *ACTB*, *SDHA* and *YWHAZ* were designed using Primer5 software based on bovine genomic sequences available in GenBank, others (*GAPDH*, *HPRT1, TATABP)* were adopted from previous reports (9, 29) (**Table 1**).

**Table 1:** Primer sequence and biological function of the employed reference genes.

Gene name	Gene symbol	Biological function	Primers sequence	GeneBank accession number	Amplicon length [bp]	Melting temperature [°C]	References
β-actin	*ACTB*	structural protein	GAGCGGGAAATCGTCCGTGAC GTGTTGGCGTAGAGGTCCTTGC	NC_007326	278	60	designed in this study
glyceraldehyde-3-phosphate dehydrogenase	*GAPDH*	carbohydrate metabolism	ACCACTTTGGCATCGTGGAG GGGCCATCCACAGTCTTCTG	U85042	75	58	(23)
hypoxanthine phosphoribosyltransferase 1	*HPRT1*	nucleotide metabolism	TGCTGAGGATTTGGAGAAGG CAACAGGTCGGCAAAGAACT	NW_001501830	154	58	(9, 23)
succinate dehydrogenase, subunit A	*SDHA*	energy metabolism	GCAGAACCTGATGCTTTGTG CGTAGGAGAGCGTGTGCTT	NC_007318	185	60	designed in this study
TATA box-binding protein	*TBP*	transcription factor	ACAACAGCCTCCCACCCTATGC GTGGAGTCAGTCCTGTGCCGTAA	NM_001075742	111	60	(9)
tyrosine 3-monooxygenase/tryptophan 5-monooxygenase activation protein, zeta polypeptide	*YWHAZ*	lipid metabolism; cell growth and death	GCATCCCACAGACTATTTCC GCAAAGACAATGACAGACCA	NW_001493253	120	60	designed in this study

Reference gene stability in MGCLEC was evaluated using the GeNorm algorithm, accounting for Crossing Point (CP) values and the Real-Time PCR efficiency (E). Pairwise variation was determined *via* standard deviation of logarithmically-transformed CP values, while M-value represented mean transcription variability between gene pairs. A Normalization Factor (NF), calculated as the geometric mean of CPs for the two most stable reference genes, was used for relative quantification (30), with further modifications by Kościuczuk et al. (9). Relative mRNA levels were established based on reaction efficiency (E).

The qPCR procedure and the primer sequences (**Table 2**) followed those described by Kościuczuk et al. (9) for β-defensins and cathelicidin gene expression in mammary gland parenchyma with a prevalence of secretory tissue (**Table 2**). Target genes were selected based on NCBI bovine genomic sequences, with only those successfully validated on pooled bovine DNA included. Specificity was confirmed via melting curve analysis and gel electrophoresis. Genes lacking reliable amplification (e.g., *CATH1*) were excluded from the study. The analysis included: bovine β-defensin1 (enteric β-defensin - *EBD*, *DEFB1, BNBD1*), neutrophil β-defensin 4 (*BNBD4, DEFB4*), neutrophil β-defensin 5 (*BNBD5, DEFB5*), neutrophil β-defensin 10 (*BNBD10, DEFB10*), tracheal antimicrobial peptide (*TAP*), lingual antimicrobial peptide (*LAP*), cathelicidin 4 (indolicidin, *CATHL4*), cathelicidin 5 (bovine myeloid antimicrobial peptide 28, *CATHL5*, *MAP28*), and cathelicidin 6 (bovine myeloid antimicrobial peptide 27, *CATHL6*, *MAP27*). Gene nomenclature followed Uniprot (https://www.uniprot.org/) and GenBank (https://www.ncbi.nlm.nih.gov/gene/) databases.

**Table 2:** The primer sequences, amplicon length, melting temperature and GenBank accession number of the studied genes (9).

Gene name	Gene symbol	Primers sequence	GeneBank accession number	Amplicon length [bp]	Melting temperature [°C]
cathelicidin 4 (indolicidin)	*CATHL4*	ACCCATCCAATGACCAGTTTGACC TTCACTGTCCAGAAGCCCGAATCT	X67340.1	177	60
cathelicidin 5 (bovine myeloid antimicrobial peptide 28)	*CATHL5 (BMAP28)*	TCGGGAGTAACTTCGACATCACCT GGCCCACAATTCACCCAATTCTGA	X97609.1	141	60
cathelicidin 6 (bovine myeloid antimicrobial peptide 27)	*CATHL6 (BMAP27)*	ATGGGCTGGTGAAGCAATGTGTAG TGGAGTAGCGGAATGACTGGAGAA	X97608.1	163	60
β-defensin1 (enteric β-defensin)	*DEFB1 (EBD)*	ATCCTCTAAGCTGCCGTCT AGCATTTTACTGAGGGCGT	NM_175703.3	102	58
β-defensin4 (bovine neutrophil β-defensin 4)	*DEFB4 (BNBD4)*	CGTTCTTGTGCCGTGTAG AAATTTTAGACGGTGTGTTG	NM_174775	149	58
β-defensin5 (bovine neutrophil β-defensin 5)	*DEFB5 (BNBD5)*	TCCTCGTGCTCCTCTTCCTA CATATTCCAACGGCAGCTTT	NM_001130761	143	58
β-defensin10 (bovine neutrophil β-defensin 10)	*DEFB10 (BNBD10)*	AGTTATCTAAGCTGCTGGG CGCTCTGTCAAAGGGTC	NM_001115084	173	58
tracheal antimicrobial peptide	*TAP*	GCGCTCCTCTTCCTGGTCCTG GCACGTTCTGACTGGGCATTGA	NM_174776	216	57
lingual antimicrobial peptide	*LAP*	GAAATTCTCAAAGCTGCCGTA TCCTCCTGCAGCATTTTACTT	NM_203435	194	58

Quantitative PCR (qPCR) was performed using a LightCycler 480 (Roche, Mannheim, Germany) with 96-well optical plates. Each 20 μl reaction mixture contained 3μl water, 5μl of cDNA, forward and reverse primers (10 μM), 1 μl each, and SYBR Green I Master Mix 2x conc. 10 μl (Roche, Germany). The amplification protocol included a 5 min pre-incubation at 95°C, followed by 35 cycles comprising 15 s denaturation at 95°C, 30 s annealing at 58-60°C, and 20 s elongation at 72°C. A negative control (no template) was included in all runs. To verify the presence of a single gene-specific peak and the absence of primer-dimer peaks, a dissociation stage (melting curve analysis) was added. To determine PCR efficiency, a relative quantification standard curve was constructed based on a 10-fold cDNA dilution series.

### Statistical analysis

Pathogen impact on the relative gene expression was determined by analysis of variance using the MIXED procedure (SAS/STAT ver. 9.4, SAS Institute Inc., Cary, NC, USA) with the Bonferroni *post hoc* test (9). The model included random effect of animal, the fixed effect of interaction between parity with two classes (lactation 1 and 2 as the first class, and lactation 3 and 4 as the second class) and the presence or absence of bacteria in milk. This grouping was justified by preliminary analyses showing no significant differences (p>0.05) in the parameters studied between lactations 1 and 2, nor between 3 and 4. The model was as follow:

y_ijk_ = μ + a_i_ + BP_j_ + e_ijk_
were:
y_ijk_ – trait value,
μ – overall mean,
a_i_ - random effect of i-th animal (k=1, …, 29)
BP_j_ – fixed effect of the interaction between infection status and parity class (j=1, …, 6)
e_ijk_– random error.

Gene expressions are shown as the means of relative mRNA abundances with their standard errors (SE).

## Results

Major pathogens (streptococci, Gram-negative rods) were absent in 24% of quarters. Among staphylococci-positive samples, 40% contained CoPS (all identified as *S. aureus*) and 36% CoNS.

### Housekeeping genes

**Figure 1:**
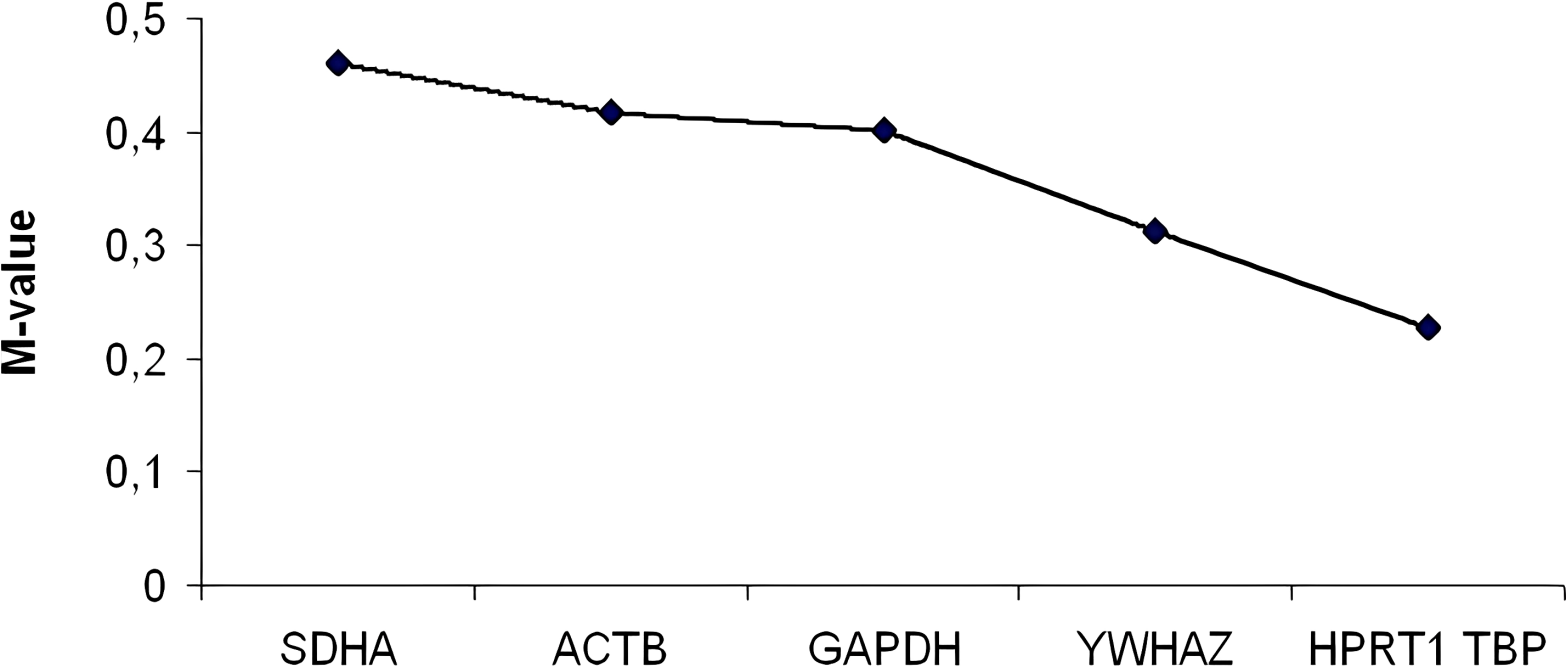
The M-values for the studied Housekeeping Genes (HKGs) in the MGCLEC.

Specificity of all HKGs was confirmed by single melting curve peaks with amplification efficiency between 92-98%. *TBP* and *HPRT1* were selected as the most stable reference genes in MGCLEC tissue, showing the lowest M-values (below 0.3; **Figure 1**). Other candidate genes also remained within accepted limits l (<0.6).

### Expression of the studied genes

**Figure 2:**
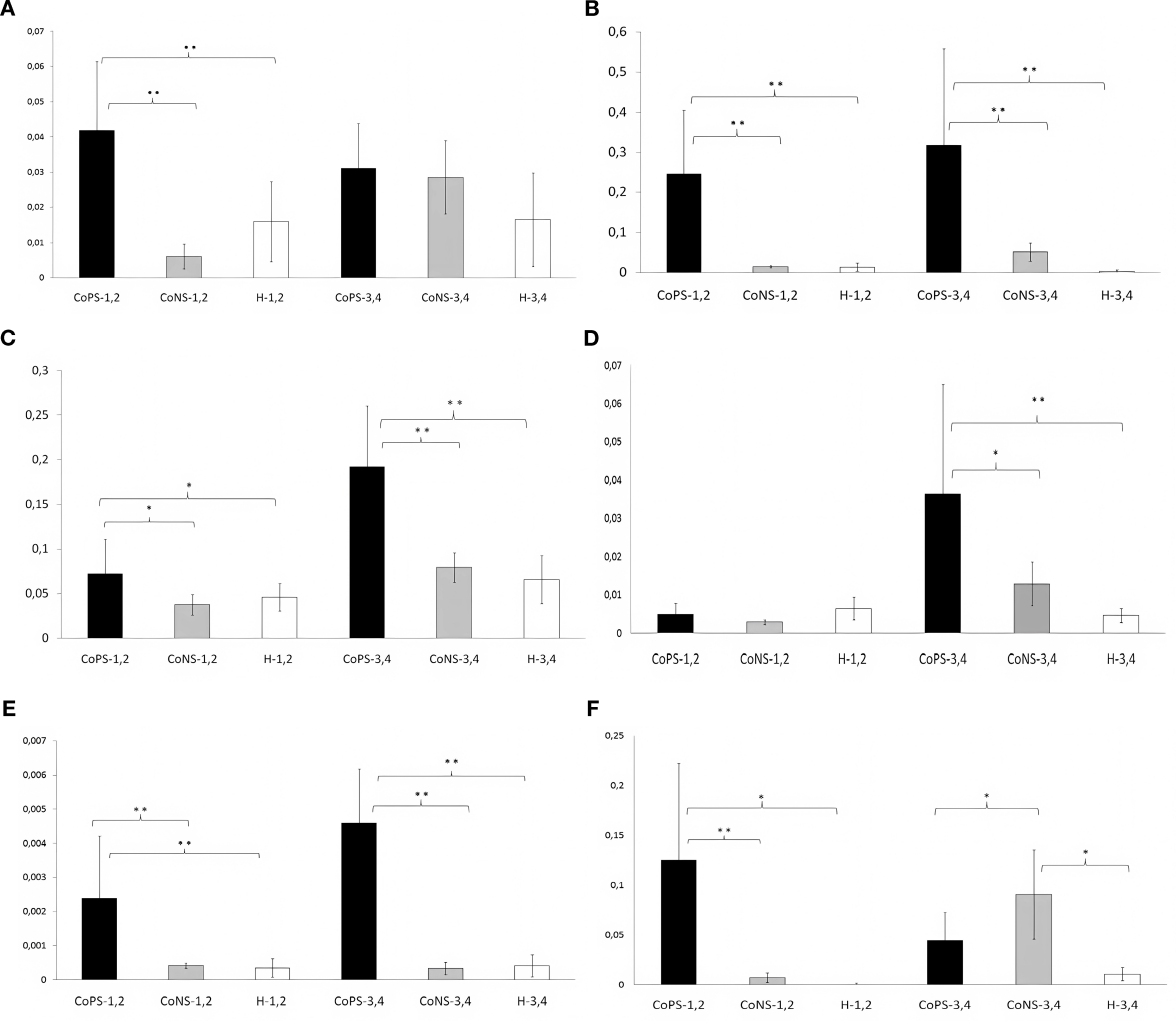
**(A)** The relative mRNA expression *of BNBD1* in MGCLEC; B. The relative mRNA expression *of BNBD4* in MGCLEC; **(C)** The relative mRNA expression *of BNBD5* in MGCLEC; **(D)** The relative mRNA expression *of BNBD10* in MGCLEC. **(E)** The relative mRNA expression *of LAP* in MGCLEC. **(F)** The relative mRNA expression *of CATHL5* in MGCLEC (* - p ≤ 0.05, ** - p<0.01). CoPS – coagulase-positive staphylococci (all isolates identified as *S. aureus*); CoNS – coagulase-negative staphylococci.

*TAP*, *CATHL4* or *CATHL6* mRNA remained undetected in all MGCLEC samples. For other genes, infection status and parity interaction was significant (p ≤ 0.05). Although *CATH5* and remaining defensins were present, their levels were notably low in the CoNS and H groups (**Figures 2A–F**). *BNBD1* levels showed a significant interaction between infection status and parity (p ≤ 0.05). In cows in lactations 1 or 2, *BNBD1* expression was higher in the CoPS-1, 2 group than in CoNS-1, 2 and H-1, 2 (p<0.01; **Figure 2A**) with no difference between the latter two (p>0.05). Older cows (lactations 3-4) showed no differences regardless of infection status (p>0.05). Overall, *BNBD1* mRNA level remained low (p ≤ 0.05) across all groups.

*BNBD4* also showed a significant infection × parity interaction (p ≤ 0.05). In both parity classes, CoPS (*S. aureus*) groups exhibited the highest transcript levels (**Figure 2B**), significantly exceeding those in CoNS and H quarters (p ≤ 0.05 for lactations 1-2; p<0.01 for lactations 3 -4.

*BNBD5* also showed a significant infection × parity interaction (p<0.01). In both parity classes, *BNBD5* mRNA was higher in the CoPS (*S. aureus*) group than in CoNS and H (p<0.01; **Figure 2C**). No differences were found between CoNS and H (p>0.05).

*BNBD10* also showed a significant infection × parity interaction (p ≤ 0.05). Unlike *BNBD4/5*, differences occurred only in older cows (lactations 3–4), where CoPS levels exceeded CoNS and H (p<0.01; **Figure 2D**). No differences were found in younger cows.

*LAP* also showed a significant infection × parity interaction (p ≤ 0.05). In both parity classes, LAP levels were higher in CoPS than in CoNS and H (p<0.01; **Figure 2E**), with no differences between the latter two (p>0.05).

*CATH5* also showed a significant infection × parity interaction (p ≤ 0.05). In first-parity cows, *CATH5* was higher in CoPS-1, 2 than in CoNS-1, 2 (p≤0.01) and H-1, 2 (p ≤ 0.05; **Figure 2F**). Conversely, in older cows, CoNS-3, 4 differed from both CoPS-3, 4 and H-3, 4 (p ≤ 0.05), with no difference between the latter two.

## Discussion

Our findings demonstrate that intramammary infection modulates cathelicidins and defensins mRNA expression, with both upregulated and downregulated transcription being noted in infected tissue. The transcriptional profile was influenced by the type of pathogen and the parity of the cow. This suggests that the immune response in the mammary cistern is uniquely shaped by bacterial pathogenicity and lactation stage.

*TAP, CATHL4*, and *CATHL6* were not detected in both CoPS (*S. aureus*) or CoNS-infected samples suggesting that these peptides are not a part of the sustained response to chronic subclinical staphylococcal infections in MGCLEC. This absence may reflect transient expression or dependence on pathogen load rather than a lack of biological role. Absence in pathogen-free quarters confirms that they are not constitutively active in MGCLEC. Interestingly, while TAP was also absent in previous studies of mammary gland parenchyma (9), CATHL4 and CATHL6 were present there, suggesting compartment-specific expression. Therefore, our results should be interpreted with caution, as these peptides might be regulated at the post-transcriptional level or expressed only under specific clinical conditions. Thus, our findings need further evaluation at the protein level to better understand the influence of post-transcriptional regulation (31).

In contrast, basal *BNBD1*, *BNBD4*, *BNBD5, BNBD10, LAP*, and *CATHL5* expression in healthy samples suggests constitutive activity in MGCLEC. Their upregulation in CoPS (*S. aureus*) groups confirms a role in defense against staphylococci, mirroring pathway in udder parenchyma (9). Thus, MGCLECs function not only as a mechanical barriers but also as active compound of the innate immune response *via* antimicrobial peptides transcription.

Our findings are consistent with those of Whelehan et al. (16), who found induced *BNBD4* and *BNBD5* gene expression in MGCLEC from *S. aureus* challenged quarters, but only partially with those of Tetens et al. (14). Unlike Tetens et al. (14), we found no *TAP* transcripts in healthy tissues and notably lower *LAP* expression. Discrepancies also exist with Petzl et al. (8, 32) who reported delayed *BNBD5* expression and stable *LAP* levels in the teat cistern, likely due to differing udder tissue types. Conversely, our findings that *LAP* increases during infection (both CoPS and CoNS) correlates with Swanson et al. (11), linking expression to higher SCC in milk.

Discrepancies between our findings and previous studies are likely attributable to variations in infection duration and tissue type (8, 16, 32). Unlike experimental models, which induce acute and rapid immune responses, our study analyzed naturally occurring subclinical infections *in vivo*, making direct comparisons challenging but offering more representative field-level data.

Contrary to our findings, chronic *S. aureus* inflammation was previously linked to elevated *LAP* and *TAP* transcript levels in MGCLEC, with higher *TAP* expression compared to parenchymal tissue (33). However, our results align with Whelehan et al. (16), who also reported no significant *BNBD1*, *LAP*, and *TAP* induction in healthy quarters.

Roosen et al. (15) identified *LAP, TAP*, and *BNBD1* in various lactating and non-lactating tissues. However, direct comparison with our results is limited, as their study encompassed multiple mastitis forms without distinguishing between specific tissue compartments.

Defensin expression has also been studied in milk somatic cells derived from mastitic udders. Neumann et al. (34) reported elevated DEFB4 levels in clinical mastitis, *vs.* subclinical mastitis. Kawai et al. (35), observed higher LAP concentrations in infected milk. Similarly, Ogbebor (36) found increased concentration of BNBD4 and LAP protein levels in clinical cases However, comparing these protein data with our mRNA results requires caution due to post-transcriptional regulation, miRNA activity, or varying transcript stability. Consequently, mRNA levels do not always reflect protein abundance in the same tissue (37–39).

Consistent with our findings, constitutive expressions of *BNBD1*, *BNBD4, BNBD5*, and *LAP* occurs in bovine tissues other than the mammary gland including lung alveolar macrophages (40), distal part of the small intestine (41), and tongue and tracheal epithelia (42, 43), supporting their role in mucosal defense.

Data on cathelicidins in the bovine mammary gland, particularly in MGCLEC remains limited. Previously, *CATH4*, *CATH5* and *CATH6* transcripts were identified in bovine mammary gland parenchyma with a prevalence of secretory tissue, across all infectious statuses and parities (9), while expression was generally highest in the H group, *CATH5* was elevated in samples derived from their 3–4 lactations. Whereas *CATH6* levels were higher in younger cows (lactations 1–2), particularly in the CoNS group (9).

Elevated cathelicidin expression in non-infected tissue suggests role in maintaining mammary gland homeostasis. While Addis et al. (44) detected cathelicidins in mastitis milk, (both *S. aureus* and CoNS), their pan-cathelicidin sandwich ELISA test with monoclonal antibodies covering regions of identity between 99% and 68% to CATH1–7 could not distinguish individual peptides (identity of 99% with CATHLA1_BOVIN, 72% with CATHLA2_BOVIN, 69% with CATHLA3_BOVIN, 68% with CATHLA4_BOVIN, 80% with CATHLA5_BOVIN, 73% with CATHLA6_BOVIN and 73% with CATHLA7_BOVIN). This complicates direct comparisons with our gene-specific mRNA data.

Katsafadou et al. (45) reported Cath1 absence in non-inoculated sheep udder during *Mannheimia haemolytica* and *Staphylococcus chromogenes* challenge. Although Cath1 was not investigated in the present study, its role in mammary defense warrants further study.

Cathelicidins 1–7 have also been identified in bovine endometrial epithelial cells, with protein levels rising during moderate endometritis, i.e. in endometrial tissue challenged with *E. coli (*46*)*,. Similarly, the cathelicidin *CAP18* (also known as *FALL18*) is expressed in the epithelia of various organs, such as the respiratory or gastrointestinal tracts of *Rhesus macaque* (*Macacamulatta*) (47). As epithelial tissue in various glands, including the mammary gland, serves both secretory and protective function, cathelicidins likely play a crucial role in the innate immunity of the cattle mammary gland. However, further studies are needed to fully establish their functional significance in the mammary gland under both physiological or pathological conditions.

As noted by Gurao et al. (48), the first line of defense in the mammary gland is the physical barrier provided by the epithelial tissue. When combined with defensins and other AMPs, this barrier is capable of preventing intramammary infection or even clear pathogens from the udders before a full inflammatory response is initiated. Only after the initial physical and chemical defense is breached does the system trigger a broader immune response activating resident macrophages and recruiting neutrophils to the site of infection. Thus, the MGCLEC plays a dual role in udder defense, acting both as a robust mechanical barrier and as a crucial source of innate immune factors.

Since the bilayer epithelium of the mammary gland lacks a protective mucus layer, MGCLEC are directly exposed to any bacteria passing through the teat canal. To counteract this threat, the mammary gland employs inflammation to control bacterial proliferation within the lumen and along the epithelial lining. Consequently, neutrophil recruitment must respond rapidly to such challenges, requiring a low activation threshold within the epithelial tissue (49).

Previous studies reported the highest expression of *LAP* and *TAP* within the lactiferous sinus and glandular tissue (33); suggesting that their levels increase as pathogens become established in these regions. This potentially indicates a role of LAP and TAP in the local immune response during persistent infections, such as those caused by *S. aureus*. and that the mammary gland may experience chronic inflammation without eliminating the *S aureus*. However, the notably low or absent expression of these specific genes in our study suggests that their induction may depend on the particular stage or severity of the infection.

Comparing our results with previous research remains challenging, as most studies on mastitis focus on milk, blood, or udder parenchyma, with a prevalence of secretory tissue. Investigations specifically targeting MGCLEC are rare; however, it is crucial to determine whether MGCLEC serves only as a physical barrier against pathogens or play a more complex role in the propagation of intramammary infection.

## Conclusion

Our results suggest that *BNBD4*, *BNBD5*, and *CATHL5* participate in the MGCLEC defense against staphylococci, given their transcriptional response to CoPS (*S. aureus*). Their activation could potentially limit the spread of infection to deeper regions of the mammary gland. Furthermore, the consistent detection of four β-defensins and one cathelicidin in both healthy and infected samples indicates they are constitutively expressed in this tissue. In contrast, the absence of *TAP*, *CATHL4*, or *CATHL6* mRNA suggests these specific peptides might not be the primary transcriptional defense factors against subclinical staphylococcal mastitis in MGCLECs. As our study is based on RT-qPCR, further research using protein-level analyses, such as ELISA or immunohistochemistry, is necessary to determine if protein abundance correlates with our transcriptomic data.

## Data Availability

The data are deposited at https://doi.org/10.58132/FVVEKB and are publicly available.
